# Comprehensive Mutational and Phenotypic Characterization of New Metastatic Cutaneous Squamous Cell Carcinoma Cell Lines Reveal Novel Drug Susceptibilities

**DOI:** 10.3390/ijms21249536

**Published:** 2020-12-15

**Authors:** Jay Perry, Bruce Ashford, Amarinder Singh Thind, Marie-Emilie Gauthier, Elahe Minaei, Gretel Major, Narayanan Gopalakrishna Iyer, Ruta Gupta, Jonathan Clark, Marie Ranson

**Affiliations:** 1 Molecular Horizons and School of Chemistry and Molecular Bioscience, University of Wollongong, Wollongong, NSW 2522, Australia; jrp998@uowmail.edu.au (J.P.); bgashford@gmail.com (B.A.); eminaei@uow.edu.au (E.M.); gretel.major@gmail.com (G.M.); 2 Illawarra Health & Medical Research Institute, Wollongong, NSW 2522, Australia; athind@uow.edu.au; 3 CONCERT Translational Cancer Research Centre, Liverpool, NSW 2170, Australia; 4 Sydney Head and Neck Cancer Institute, Chris O’Brien Lifehouse, Camperdown, NSW 2050, Australia; ruta.gupta@health.nsw.gov.au (R.G.); jonathan.clark@lh.org.au (J.C.); 5 School of Medicine, University of Wollongong, Wollongong, NSW 2522, Australia; 6 Kinghorn Centre for Clinical Genomics, Garvan Institute of Medical Research, Darlinghurst, NSW 2010, Australia; maelygauthier@gmail.com; 7 Department of Surgical Oncology, National Cancer Centre Singapore, Singapore 169610, Singapore; gopaliyer@yahoo.com; 8 Department of Tissue Pathology and Diagnostic Oncology, Royal Prince Alfred Hospital, NSW Health Pathology, Camperdown, NSW 2050, Australia

**Keywords:** cSCC, metastasis, skin cancer, PI3K, WGS, ultraviolet, cancer, cell culture, xenograft, gene expression

## Abstract

Cutaneous squamous cell carcinoma (cSCC) is a common skin cancer. Most patients who develop metastases (2–5%) present with advanced disease that requires a combination of radical surgery and adjuvant radiation therapy. There are few effective therapies for refractory disease. In this study, we describe novel patient-derived cell lines from cSCC metastases of the head and neck (designated UW-CSCC1 and UW-CSCC2). The cell lines genotypically and phenotypically resembled the original patient tumor and were tumorogenic in mice. Differences in cancer-related gene expression between the tumor and cell lines after various culturing conditions could be largely reversed by xenografting and reculturing. The novel drug susceptibilities of UW-CSCC1 and an irradiated subclone UW-CSCC1-R to drugs targeting cell cycle, PI3K/AKT/mTOR, and DNA damage pathways were observed using high-throughput anti-cancer and kinase-inhibitor compound libraries, which correlate with either copy number variations, targetable mutations and/or the upregulation of gene expression. A secondary screen of top hits in all three cell lines including *PIK3CA*-targeting drugs supports the utility of targeting the PI3K/AKT/mTOR pathway in this disease. UW-CSCC cell lines are thus useful preclinical models for determining targetable pathways and candidate therapeutics.

## 1. Introduction

Cutaneous squamous cell carcinoma (cSCC) is a common non-melanoma skin cancer and the most common malignancy worldwide [1,2]. Primary cSCC is typically treatable, although in 2–5% of cases metastatic spread occurs [3,4,5,6,7,8], resulting in the majority of disease-specific deaths. Other than surgery and radiotherapy, the only systemic therapy approved for locally advanced and metastatic cSCC is Cemiplimab immunotherapy, which resulted in a 50% response rate and was associated with adverse events [9,10].

Recent efforts are helping to identify the biological processes underpinning the tumorigenesis and metastasis of cSCC [11,12,13,14,15,16,17,18,19]. However, candidate biomarkers and therapeutics have seldom been examined in pre-clinical models of metastatic disease. Cell lines remain powerful pre-clinical tools to study biological behavior, as well as being amenable to high-throughput drug screening [20,21,22]. Cell lines derived from primary cSCC have been reported [23,24,25,26,27,28], but there are few cell lines derived from metastatic cSCC (Table S1). The metastatic cell lines UT-SCC7, UT-SCC59A, and UT-SCC115 have not been validated molecularly, nor has evidence of tumorigenicity been published. This is also the case for an undesignated metastatic cSCC cell line recently developed [29]. MET4 and IC1MET form tumors in mice but phenotypic comparisons to their originating tumors are limited. The mutational profile of IC1MET was, however, analyzed by whole-exome sequencing and shown to be comparable to its originating tumors [12]. Regardless, there is a need for additional high-fidelity models of metastatic cSCC to cover the mutational spectrum observed between patients.

In this current study, we report the establishment of novel patient-derived cSCC cell cultures from nodal metastases, designated UW-CSCC1 and UW-CSCC2. The originating tumors and in vitro derivatives underwent whole-genome sequencing (WGS), gene expression analysis, and other phenotypic analyses to characterize the fidelity of the cell lines and the implications of these findings for therapeutic investigations. The benefits of these cell lines are shown through high-throughput screening of anti-cancer molecules, revealing candidate therapeutics for this disease.

## 2. Results

### 2.1. Phenotypic Validation of Novel Cell Lines from Patients with Metastatic cSCC

**Figure 1:**
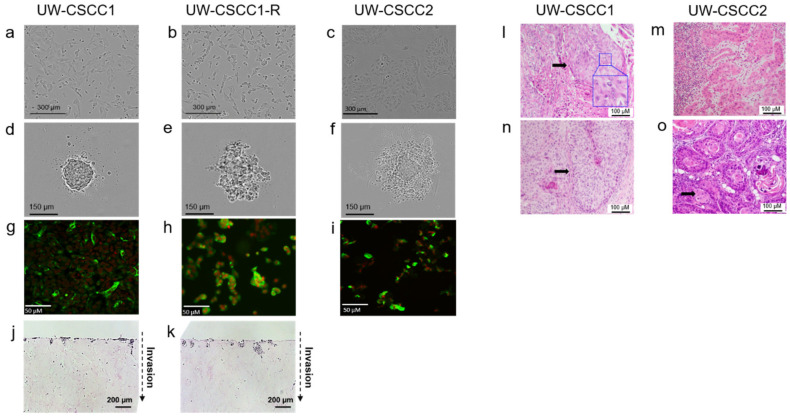
Tumor and cell line characterization by microscopy. Photomicrographs (10× objective) of cell lines grown in (**a**–**c**) monolayer (2D) culture or (**d**–**f**) spheroid (3D) cultures. Immunocytochemical images (**g**–**i**) of cell lines stained with CK-5 antibody (green): nuclei stained with RedDot2 (red). Organotypic assays showing UW-CSCC1 (**j**) and UW-CSCC1-R (**k**) directional invasion into fibroblast contracted collagen I matrices at eight days after being placed onto an air–liquid interface. Representative micrographs of hematoxylin and eosin (H&E)-stained sections (*n* = 3 matrices). Magnification 20× objective. Dotted—Patient 1 and (**n**) UW-CSCC1 xenotransplant in NSG mouse or (**m**) clinical tumor from Patient 2 and (**o**) UW-CSCC2 xenotransplant in NSG mouse. Enlarged inset panel (**l**) highlights polyploidy. Arrow heads denote papilliform architecture.

Long-term continuous cell lines were established from parotid node metastases from two patients (Table S2), designated UW-CSCC1 and UW-CSCC2. To model changes that may be incurred by tumor cells that survive radiation therapy, an irradiated sub-clone was expanded from passage 13 UW-CSCC1, designated UW-CSCC1-R. The doubling times of UW-CSCC1, UW-CSCC1-R, and UW-CSCC2 as monolayers were 47, 36, and 82 h, respectively. Morphologically, UW-CSCC1-R appeared more mesenchymal-like compared to UW-CSCC1 (Figure 1a,b). The UW-CSCC2 were morphologically dissimilar to UW-CSCC1/1-R, demonstrating rounder borders and a smaller diameter (Figure 1c). UW-CSCC1 and UW-CSCC2 produced tight spheres in ultralow binding plates within 48 h, whereas UW-CSCC1-R only formed aggregates (Figure 1d–f).

All cell lines stained positive for the epithelial marker CK-5 (Figure 1g–i), but were negative for the mesenchymal markers α-SMA and vimentin (data not shown), confirming their epithelial purity. UW-CSCC1 and UW-CSCC1-R demonstrated invasion using an organotypic assay (Figure 1j,k). UW-CSCC2 was not assessed in this assay. The tumorigenicity of UW-CSCC1 and UW-CSCC2 was confirmed by xenografting in NOD *scid gamma* immunocompromised mice. Tumors arose at the site of inoculation within three months and largely retained the tissue architecture of the clinical specimen (Figure 1l–o), including unequivocal squamous epithelial cell morphology and malignant cytological features (Figure 1l inset).

### 2.2. Genotypic Validation of Novel Cell Lines from Patients with Metastatic cSCC

**Figure 2:**
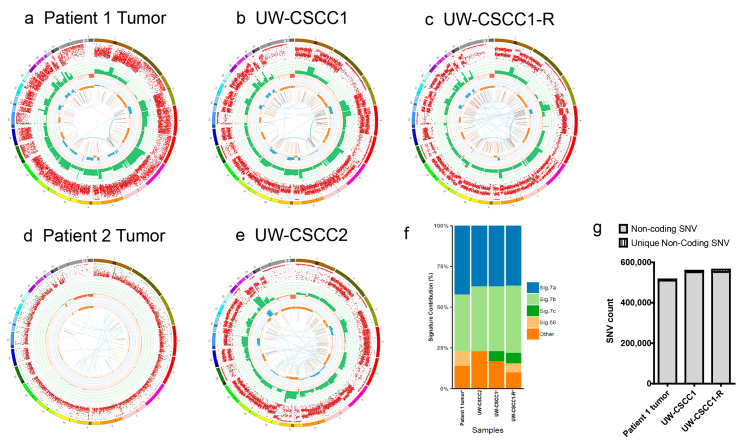
Genomic landscape of cell lines and matched tumors. Circos plots showing overall pattern of genetic aberrations between (**a**) the tumor of Patient 1 and matched cell lines, (**b**) UW-CSCC1 and (**c**) UW-CSCC1-R; or (**d**) the tumor of Patient 2 and the matched cell line (**e**) UW-CSCC2. The layers indicate the following (going from outside in): (i) chromosomes, with the darker shaded areas representing large gaps in the reference genome due to regions of centromeres, heterochromatin, and missing short arms; (ii) the purity-adjusted allelic frequency of all observed SNV (including introns and intergenic regions); (iii) all observed copy number changes; with losses indicated in red and copy number gain shown in green; (iv) the minor allele copy number (minor allele losses are indicated in orange, whilst blue shows regions of minor allele gain); (v) the observed structural variants within or between the chromosomes (translocations are indicated in blue, deletions in red, insertions in yellow, tandem duplications in green and inversions in black). (**f**) Mutational signature frequency of cell lines and tumor from Patient 1. (**g**) Total number of non-coding variants detected amongst patient 1 tumor, UW-CSCC1, and UW-CSCC1-R. The unique non-coding SNV specific to each sample is overlain.

The genomic characteristics of parent tumors and cell lines analyzed by whole genome sequencing are summarized in Figure 2a–e. The low levels of variant detection for the Patient 2 tumor (Figure 2d) were likely a result of the low tumor cellularity of the sample taken for DNA extraction, as confirmed after WGS (14% by Purple analysis; Table S2), and thus could not be compared genomically to its corresponding cell line UW-CSCC2. Major structural variation was present in UW-CSCC2, including deletions, amplifications, inversions and translocations (Figure 2e), similar to that observed for the Patient 1 tumor and its derived cell line (Figure 2a–c).

The structural variation patterns (copy number gains and losses and minor allele copy number) were well conserved between UW-CSCC1/1-R and the originating tumor (Figure 2a–c), with the exception of some UW-CSCC1-R-specific reductions in minor allele copy number present in chromosomes 9 and 11. The UV-associated (C > T) mutational signature was conserved in the cell lines (Figure 2f), accounting for >70% of small nucleotide variants (SNVs) across all samples. Some discordance was evident with respect to signature 7c and signature 58 between the various cell lines and Patient 1 tumor.

The numbers of SNVs in the non-coding regions were similar between the samples, despite some additional variants in the cell lines (Figure 2g). The up- and down-stream 2 kbp regions were analyzed more discretely, revealing a similar pattern of fidelity (Figure S1), which has implications for gene transcription. A detailed analysis of the coding variants in the Patient 1 tumor, its associated cell lines and UW-CSCC2 identified 6518 exonic SNVs across 4335 genes (Figure S2a,b). Most of these mutations functionally relate to poly(A) RNA binding (Figure S2c). In the Patient 1 tumor and related cell lines, a substantially higher number of variants was observed compared to UW-CSCC2. Of the 2992 genes featuring exonic variants in the Patient 1 tumor, 2924 (97.73%) were shared with matched cell lines UW-CSCC1 and UW-CSCC1-R (Figure S2d). A further 165 affected genes were identified and shared between UW-CSCC1 and UW-CSCC1-R only. Either this reflects new mutations or, more likely, it demonstrates the increased purity in the cell lines, facilitating greater variant calling confidence. There were 35 and 56 genes containing exonic variants exclusive to UW-CSCC1 and UW-CSCC1-R, respectively. The majority (>97.5%) of the exclusive variants in UW-CSCC1-R were silent or missense SNVs. Others included a frameshift truncation in *PDGFRL* and a stop-gained variant in *DYNLL1* (Figure S2d). Moreover, the highest SNVs variance was observed in *AHNAK2*, *AHNAK*, *MUC12*, and *KMT2C* across the Patient 1 tumor, UW-CSCC1 and UW-CSCC1-R (Figure S2e).

**Figure 3:**
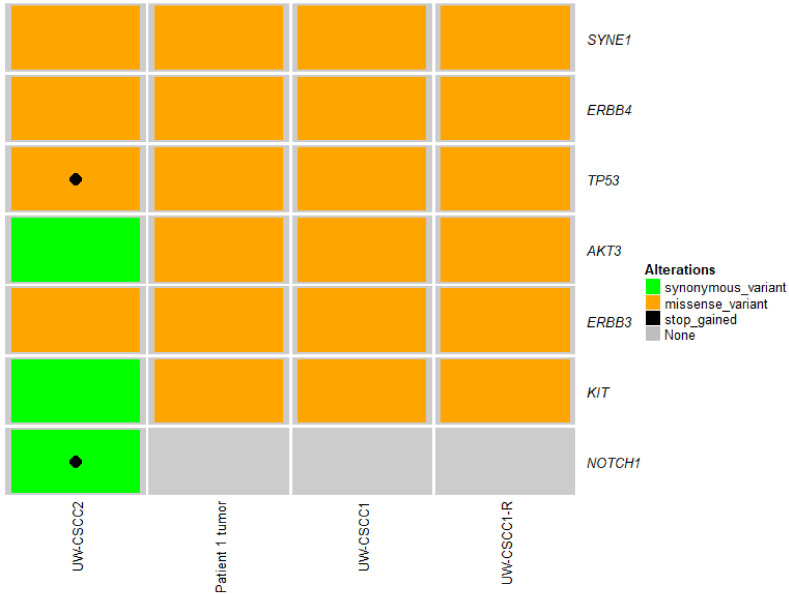
Recurrent coding short nucleotide variants of druggable targets shared across Patient 1 tumor, UW-CSCC1/-R and UW-CSCC2. Stop-gained and missense variants are considered high- and medium-impact, respectively. Synonymous variants are considered low impact. Refer to Supplementary Dataset 1 for detailed variant information.

Of the 1309 known oncogenes, tumor suppressor genes or cancer-associated genes previously examined for somatic variation in a wider group of 15 cSCC lymph node metastases, which included the parent tumor for UW-CSCC1/1-R [18], 60 genes shared short variants between UW-CSCC1/1-R and UW-CSCC2 (Table S3). Of these 60 genes, the Patient 1 tumor and its cell lines, along with UW-CSCC2, showed similar alterations across seven drug-targetable genes (Figure 3). Not surprisingly, *TP53* inactivating mutations were common across these cell lines (and the lymph node metastases, Table S3), suggesting susceptibility to DNA-damaging drugs. While *NOTCH1* mutations often occur concomitantly with *TP53* in cSCC [30,31], this was not evident in the Patient 1 tumor and its derivative cell lines (Figure 3). Further, the Patient 1 tumor, its derivative cell lines and UW-CSCC2 all harbored copy number amplifications in genes of the PI3K/mTOR signaling pathway (Table S4), as well as mutations in *AKT3* (Figure 3), supporting this as a key targetable pathway in metastatic cSCC. Altogether, this suggests that as these cell lines share several targetable alterations in cancer driver genes, they are thus broadly representative of the metastatic cSCC of the head and neck in the context of drug susceptibility testing.

### 2.3. Effect of In Vitro Culture on Cancer Gene Expression Patterns

**Figure 4:**
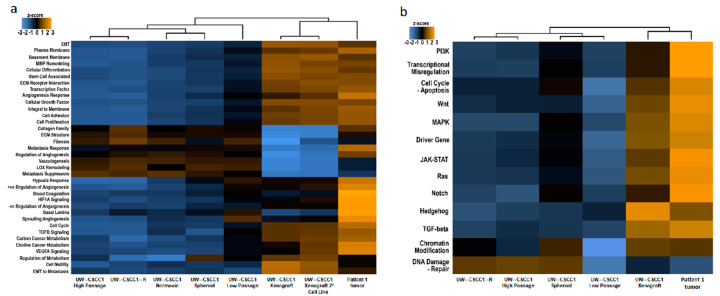
Cancer gene expression analysis. (**a**) nCounter PanCancer progression and (**b**) nCounter Pancancer pathways panel gene expression pathway score profiles for Patient 1 and UW-CSCC1 culture derivatives. Nanostring heatmaps showing how pathway scores (fit using the first principal component of each gene set’s data) change across samples or culture conditions. Pathway scores condense each sample’s gene expression profile into a small set of pathway scores. Orange indicates high scores; blue indicates low scores, i.e., samples that exhibit similar pathway score profiles. Scores are displayed on the same scale via a Z-transformation. Sample details including passage number are provided in Table S5.

The unbiased clustering analysis of gene expression from the nCounter panels separated the Patient 1 tumor-derived in vitro cell lines from in vivo (patient tumor and xenograft) samples (Figure 4). Of the various in vitro conditions tested, passage number resulted in the most impact, with low passage number separating UW-CSCC1 away from the cells under the other in vitro conditions (i.e., high passage, irradiated, normoxic and spheroid), which otherwise exhibited very similar pathway score profiles. Indicators of stemness and DNA damage repair were upregulated in UW-CSCC1-R relative to UW-CSCC1 (Figure S3a,b). Cell adhesion gene set expression was mostly downregulated in UW-CSCC1-R (Figure S3c), which was reflected physiologically in the inability of UW-CSCC1-R to form tight spheroids compared to UW-CSCC1.

Many pathways’ gene set expressions under in vitro conditions were downregulated relative to the in vivo samples, with differences between xenograft samples and the Patient 1 tumor evident only for a subset of pathways in the PanCancer progression analysis (Figure 4a). In the PanCancer pathways analysis, the gene set expression in all biological categories was very similar between the xenograft and the Patient 1 tumor (Figure 4b). A comparison of the overall gene expression of a very early passage of xenograft-derived UW-CSCC1 (Xenograft 2° UW-CSCC1 cell line) to the xenograft (Figure 4a) revealed similar pathway score profiles.

### 2.4. High-Throughput and Secondary Drug Screens Reveal Novel Therapeutic Targets in Metastatic cSCC Cell Lines

**Figure 5:**
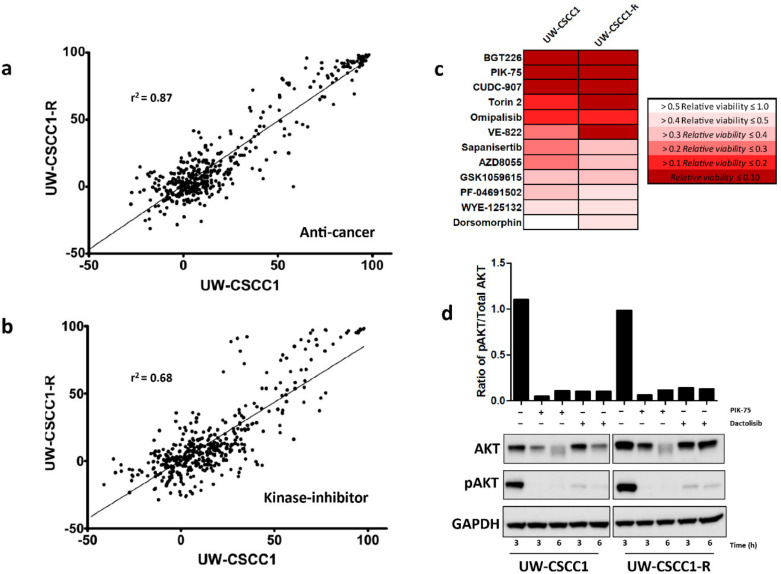
Drug screen response of UW-CSSC1/1-R. Overall distribution of percentage inhibition is shown for the anti-cancer (**a**) and kinase-inhibitor (**b**) libraries. Negative inhibition implies a pro-proliferative event has occurred. Lines of best fit and coefficients of determination are shown. (**c**) Heatmap showing the relative viability effect on the cell lines of the topmost effective PI3K/AKT/mTOR inhibitors. (**d**) Western blots demonstrating total AKT and phosphorylated AKT (pAKT) levels in response to pretreatment with the respective IC_50_ value of the PI3K inhibitors PIK-75 or Dactolosib for the times shown for both cell lines. The housekeeping gene GAPDH was used as a total protein loading control. The densitometry analysis shows the ratios of pAKT/total AKT for each treatment.

The novel drug susceptibilities of UW-CSCC1 and UW-CSCC1-R were identified using single-dose anti-cancer compounds and kinase inhibitor screening libraries. The top 40 most efficacious drugs for both libraries, along with their respective targets, are listed in Tables S5.1–S5.4. Both libraries demonstrated a bimodal distribution, partitioning the majority of tested compounds as either ineffective or highly effective at 1 µM (Figure 5a,b). Between the cell lines there was a strong and moderate positive correlation for the anti-cancer and anti-kinase drug response, respectively. The majority of the effective drugs (with > 70% inhibition) in the anti-cancer library targeted cell cycle, cytoskeletal signaling, DNA damage, as well as protease pathways (Tables S6.1–S6.2). Efficacious compounds included topoisomerase or cyclin-dependent kinase (CDK) inhibitors. The highly efficacious compounds in the kinase-inhibitor library largely pertained to targeting the cell cycle (particularly via CDKs), the cytoskeleton, or the PI3K/Akt/mTOR pathways (Tables S6.3–S6.4) in both cell lines.

Given the response to the PI3K inhibitor PIK-75 and the associated gene alterations in the PI3K/AKT/mTOR pathway in all three cell lines, a secondary dose-response screen was performed to elucidate IC_50_ values. The UW-CSCC1 and UW-CSCC1-R sensitivity to PIK-75 did not differ substantially, with IC_50_ values being 0.122 ± 0.012 µM and 0.204 ± 0.05 µM, respectively. PIK-75 was more potent against UW-CSCC2 compared to the other cell lines, exhibiting an IC_50_ of 0.026 ± 0.012 µM. The dual PI3K/mTOR-targeting compound dactolisib was also strongly potent against the cell lines. The inhibition of pAKT was observed with UW-CSCC1 and UW-CSCC1-R in response to IC_50_ concentrations of PIK-75 and dactolisib, and the total AKT expression seemingly reduced in a time-dependent manner (Figure 5d). IC_50_ values for other common chemotherapeutics against the cell lines are provided in Table 1 for comparison. Of note, UW-CSCC2 appears to be more resistant than UW-SCC1/1-R to carboplatin, which may reflect its dual *TP53*/*NOTCH1* high impact mutations (refer to Figure 3).

## 3. Discussion

This is the first study to apply WGS and expression analysis on metastatic cSCC cell lines, and to correlate those findings with small molecule drug responsiveness. Our results show that the C > T predominance of cSCC tumors and UV-associated mutation signature 7 are retained in the cell lines. UW-CSCC1 almost completely retained the mutational landscape of the clinical tumor, as has been witnessed in other cell lines [32], suggesting that genetic drift is minimal following 2D culture. The additional coding and non-coding variants called in UW-CSCC1/1-R are not explicit evidence of genetic drift, but rather a result of the culture purity enhancing variant detection through the elimination of the stromal background. Adjustments in copy number were to be expected given that they are generally seen to be in greater numbers in cell lines [20]. The identification of SNVs and CNVs among the cell lines pertaining to the PI3K/AKT/mTOR pathway builds upon previous observations [33,34,35], supporting its prognostic utility, as well as being an indicator of therapeutic responsiveness to corresponding selective inhibitors, as assessed by our compound screening.

The downregulated cancer gene expression profiles observed in the cell lines compared to the original tumor are unsurprising given this effect has been seen in many cell lines of other cancer types [36,37,38]. Contrastingly, Barretina et al. [39] observed a strong positive correlation between 947 cell lines and primary tumors, suggesting cell lines possessed many of the genomic aberrations found in tumors. They proposed the differences between cell lines and clinical tumors are a result of background tumor microenvironment (TME)-related gene expression that could be simply subtracted, leaving purely tumor cell-associated gene signatures.

High passage cultures were found to downregulate pathways further, highlighting the vulnerability of high passage commercial cell lines. However, unpublished work from our group has shown that changes in drug responsiveness over passage numbers were subtle. The spheroid derivative demonstrated a slight change in expression profile towards the original tumor, although global changes were found to be negligible, as found with other cell types [40]. It was observed in the normoxic derivative that the genes involved in metastasis response were influenced by normoxic-inducible factors, aligning with previous literature [41], supporting hypoxic culture conditions.

Cells surviving radiation may acquire new mutations or expose previously undetected mutations present within the now-expanded subclone. The current study noted an increase in *PCNA*, *MAD2L2*, and *FEN1* gene expression in UW-CSCC1-R relative to UW-CSCC1; all of which contribute majorly to DNA damage repair [42,43,44]. In addition, cell adhesion and stem cell-associated genes were also altered in UW-CSCC1-R, contributing to a more mesenchymal physiology and likely contributing to the changes observed in proliferation and drug responsiveness. In a clinical context, this may result in more aggressive local recurrence; therefore, continued investigation into pre-clinical models based on UW-CSCC1-R that imitate this scenario are warranted.

The cSCC secondary xenograft mostly regained the original tumor phenotype (indicative of genome stability), with the exception of the following pathways: collagen family, ECM structure, metastasis response, and regulation of angiogenesis. This may be due to the mouse stroma inhibiting transcription of the relevant genes in the human cells, or species incompatibility as murine growth factors do not activate certain human-specific pathways, e.g., human MET [20,22]. A similar study compared cell lines with their successive xenografts and found a number of gene classes becoming enriched in the xenograft tumors [45]. In contrast, it has been observed for small cell lung cancer that many of the changes incurred by going onto plastic were irreversible [46]. We infer that this restorative ability is cell line-dependent.

Thus, altogether, the WGS and gene expression data indicate that genotype was preserved in the cell lines and that differences in gene expression profiles due to in vitro culture can be largely restored in vivo. This property permits the highly reproducible and cost-effective derivation of cell lines in vitro for efficient biological assays, with the capability of resembling in situ tumor biology, given the appropriate environment [45].

The establishment and anti-cancer drug library screening of UW-CSCC1 and its derivatives also revealed efficacious agents and drug classes that will draw our focus in future investigations. The moderate correlation in drug sensitivity between UW-CSCC1 and UW-CSCC1-R highlights the effect that irradiation can have upon drug response. Nonetheless, the cells are still quite responsive to chemotherapy post-radiation which, for the case in vivo, implies the potential benefit for chemotherapy in the adjuvant setting or in cases of local recurrence. Drugs targeting cytoskeletal signaling were particularly efficacious and may be related to mutations in *SYNE1* which were notably present in our cell lines as well as in 14/15 of the clinical samples assessed (Table S3). This gene has a role in cytoskeletal arrangement and has been proposed as a biomarker of colorectal cancer in liquid biopsies [47]. Dose-response screening and western blot analysis verified the sensitivity of the cell lines towards PIK-75, as well as the dual mTOR/PI3K-targeting dactolisib, thus permitting further pre-clinical studies on the PI3K/AKT/mTOR oncogenic pathway in metastatic cSCC. A recent study similarly identified sensitivity to dactolisib for invasive cell lines of cSCC [29]. According to the Genomics of Drug Sensitivity in Cancer Database (https://www.cancerrxgene.org/; Accessed 1 December 2020) the mean IC_50_ for head and neck SCC cell lines using dactolisib (PIK-75 data not available) was notably higher (311 nM) than the range observed with our cell lines (22–80 nM), further supporting the sensitivity of our cell lines to this drug. Whilst cell cycle and cytoskeleton-targeting drugs were also unsurprisingly highly efficacious in our study [48], these compounds do not offer the same degree of specificity as PI3K/AKT/mTOR-targeting agents. Interestingly, some PI3K inhibitors produced little to no response against the cell lines, a likely result of isoform specificity that requires further investigation [33]. The anti-EGFR biologic Cetuximab is sometimes given to patients with advanced cSCC, achieving overall response rates approaching 50% [49]. However, there are multiple mechanisms by which downstream effectors of EGFR may become activated, including via PI3K [50]. Therefore, the continued examination of such pathways in advanced cSCC is necessary.

Not surprisingly, *TP53* inactivating mutations were common across these cell lines and the clinical specimens assessed, which may explain their sensitivity to DNA-damaging drugs. The direct target of p53, *NOTCH1*, is also often disrupted in cSCC and generally occurs early in cSCC [51]. However, the Patient 1 tumor and its derivative cells lines did not harbor *NOTCH1* mutations, suggesting this is not a universal feature required for metastases. Concomitant *NOTCH1* and *TP53* stop-gain mutations (high impact loss of function mutations) in UW-CSCC2 may explain the cell line’s resistance towards carboplatin relative to UW-CSCC1 and UW-CSCC1-R.

In conclusion, the generation of the UW-CSCC cell lines with detailed genomic characterization and HTS drug-screen data has provided important new insights into the biology of metastatic cSCC and revealed new candidate therapeutic targets for this disease. Whilst much of the transcriptomic difference noted may prove experimentally and clinically irrelevant, caution must be exercised when undertaking assays dependent upon the mechanisms provided by any significantly altered genes and/or pathways. However, these differences may be negated once they are re-established in vivo. It is hoped that the provision and validation of these novel cell lines will stimulate further investigations in this field.

## 4. Materials and Methods

### 4.1. Patient Characteristics and Specimen Selection

An intraparotid lymph-node metastasis of cSCC was resected from a 73-year-old male (See Table S2 for patient characteristics). Clinical staging of the tumor was performed in accordance with the 8th edition of the American Joint Committee on Cancer (AJCC) TNM staging manual. An area of the tumor sample with 70% neoplastic content without necrosis, haemorrhage, high keratin content or significant inflammation was selected for cell culture and nucleic acid extraction. A parotid nodal metastasis from another patient was also processed (86-year-old male), however this sample forwent extensive genomic and phenotypic analysis for reasons noted below. Tissue and blood were obtained with written informed patient consent in accordance with the declaration of Helsinki and University of Wollongong Human Research Ethics Committee’s approval (HE14/397; approval date: 26 May 2015).

### 4.2. Cell Culture Development and Maintenance

Tumor samples were dissected within 1 h of surgical resection into 1 mm^3^ pieces, with some fractions of the bulk tumor mass snap-frozen in liquid nitrogen for subsequent nucleic acid extraction. Samples were either dissociated using the MACS Miltenyi human tumor dissociation kit in conjunction with the gentleMACS™ dissociator (Patient 1), or simply plated as an explant onto the tissue culture surface (Patient 2). Resultant cultures were designated UW-CSCC1 and UW-CSCC2, respectively. Dissociated tissue was passed through a 70 µm MACS SmartStrainer, the filtrate was centrifuged, and the pellet was re-suspended in Dulbecco’s Modified Eagle Medium (DMEM), supplemented with 10% heat-inactivated foetal calf serum (FCS), glucose (4500 mg/mL), and penicillin/streptomycin (50 U/mL). Explant cultures were grown in Advanced DMEM/F12, supplemented with hEGF (20 ng/mL), 1% L-glutamine, 2% FCS, and penicillin/streptomycin (50 U/mL). Cells/explants were placed into tissue culture flasks according to cell density and incubated at 37 °C under a 5% CO_2_, 3% O_2_ atmosphere.

Differential trypsinization was employed to deplete the competing fibroblast population [52]. Pure metastatic cSCC cell cultures were confirmed within 13 (UW-CSCC1) and 4 (UW-CSCC2) passages and could be frozen and thawed successfully. These cultures were henceforth regarded as low passage, whilst a high passage culture of UW-CSCC1 was also established (UW-CSCC1-high passage) following an additional 41 rounds of subculturing. To confirm the cultures as epithelial, antibodies specific to cytokeratin 4/5/6/8/10/13/18 (CK223; Abcam, Cambridge, UK, Cat # ab115974) and the epithelial cell adhesion molecule (EpCAM; Abcam, Cambridge, UK, Cat # ab7504) were used, as well as the mesenchymal marker α-smooth muscle actin (α-SMA; Abcam, Cambridge, UK, Cat # ab7817) serving as a negative control.

To obtain spheroid cultures (UW-CSCC1 Spheroid), cells were seeded in Corning^®^ Costar^®^ ultra-low binding plates (Sigma-Aldrich, St. Louis, MO, USA) at a density of 375 cells per well using the aforementioned media formulation. UW-CSCC1 cells at passage 13 were also exposed to the clinically relevant dose of 2 Gy orthovoltage x-ray radiation (Radiation Oncology Medical Physics, Prince of Wales Hospital, Sydney, Australia) and surviving radio-resistant cells expanded (UW-CSCC1-R). A normoxic acclimated population of cells (UW-CSCC1-Normoxic) was also derived by incubating under standard atmospheric oxygen levels. A list of the cell lines generated along with experimental derivations is provided in Table S5.

### 4.3. Generation of Mouse Xenografts

NOD *scid gamma* (NOD.Cg-Prkdc < scid > IL2rg < tm1Wjl >/SzJAusb) mice (aged 4–5 weeks old, Australian Bioresources, Australia) were subcutaneously inoculated with cell lines at a density of 2 × 10^6^ cells into the rear flanks. Upon reaching 10 × 10 mm^2^, tumors were harvested from the sacrificed mice for cell culture, RNA extraction, and histological analysis. For culture from xenografts, tumor tissues were cut into approximately 1 mm pieces and then incubated with tumor dissociation enzymes (Tumor dissociation kit, Miltenyi Biotec, Bergisch Gladbach, Germany) in culture media, after which the tumor homogenate was centrifuged, and the pellet was resuspended and plated in serum-free media and hypoxic conditions as above. All procedures were carried out in accordance with the Australian Code for the Care and Use of Animal for Scientific Purposes 8th edition 2013, and approved by the University of Wollongong’s Animal Ethics Committee (study AE15/17).

### 4.4. Organotypic Invasion Assay

Contractions of collagen I matrices using dermally derived telomerase induced fibroblasts (TIFs) were performed as previously described [53]. Briefly, contraction to a 3D matrix was stimulated by mixing a neutralized collagen I cocktail (8.8% *v*/*v* 10× minimal essential media (Thermo Scientific, Waltham, MA, USA); 75.8% *v*/*v* 2 mg/mL collagen I; 8% *v*/*v* 0.22 M NaOH (pH 7.4)) with TIFs (1 × 106 per 12 matrices) resuspended in FCS. The TIFs used in this assay are required to be quiescent, which was achieved by leaving the cells in culture for at least five days after confluency without a change of media. Per 35 mm petri dish (Sigma-Aldrich, St. Louis, MO, USA), 2.5 mL of the collagen–fibroblast cocktail was dispensed and allowed to polymerize for 30 min in an incubator at 37 °C. Following this, the petri dishes were topped with complete media (DMEM/10% FCS/P + S) and matrices permitted to contract over a period of 5–12 days to give a diameter no smaller than 1 cm. The media was refreshed every 2–3 days or as required depending on the state of the color indicator present within the media.

Contracted matrices were moved into 24-well plates (Greiner Bio-One, Kremsmunster, Austria) and 100 µL of complete media containing 3.0 × 105 of the cells under investigation was seeded atop each matrix. After 15 min in the incubator at 37 °C to allow the cells to settle to the matrix, a further 900 µL of media was added to each well and left to grow until confluent. The matrices were then transferred onto the top of sterile 40 mm mesh screens in 60 mm petri dishes. Fresh media was added until surface tension was created between the mesh grid and media, thereby creating an air–liquid interface to promote invasion across the chemotactic gradient (in this case the underlying media).

After 7–14 days, the matrices were fixed in 4% neutral buffered formalin for 24 h followed by another 24 h in 10% formalin. The fixed samples were dehydrated in 70% ethanol and processed overnight in an ASP200 vacuum tissue processor (Leica Biosystems, Wetzlar, Germany). Each matrix was then sliced in half dorsoventrally and embedded in paraffin using the EG1150 Modular Tissue Embedding Centre and EG1150 Cold Plate (Leica Biosystems, Germany). The resultant paraffin-embedded tissue block was sectioned at a thickness of 4 µm using a RM2255 Fully-Automated Rotary Microtome (Leica Biosystems, Germany) and transferred onto glass slides by floating sectioned tissue in a dH_2_O water-bath at 40 °C. Slides were allowed to dry overnight prior to histological staining. Sectioned tissue was deparaffinized in dipentene (POCD Healthcare, Sydney, NSW, Australia) and rehydrated using graded ethanol washes (60–100% EtOH). Hematoxylin and eosin (H&E; POCD Sciences, Australia)-staining was performed on a LeicaST4020 small linear stainer (Leica Biosystems, Germany) and slides mounted with DPX (Sigma-Aldrich, St. Louis, MO, USA). Following an overnight drying period, the invasion incurred by the cells was assessed through examination of the slides under a Leica DM4000 bright-field microscope (Leica Biosystems, Germany).

### 4.5. Nucleic Acid Extraction

Tumor and cell line DNA and RNA were extracted as was previously described (Mueller et al., 2019). All samples were quantified using the NanoDrop spectrophotometer (ND1000, ThermoFisher scientific, Waltham, MA, USA) and met the purity requirements for downstream applications (A260/280 between 1.7 and 2.3). Samples were further quality controlled prior to WGS by the sequencing facilities.

### 4.6. Gene Expression Analysis

Gene expression on the NanoString nCounter Sprint platform was assessed using total RNA (25–50 ng) with two different panels (PanCancer Progression and PanCancer Pathways; each containing 740 target and 30 “housekeeping” genes) as per the manufacturer’s instructions (NanoString Technologies, Seattle, WA, USA). Table S5 summarizes the samples run, the passage number from which extracts were made, and the panel used.

Data were analyzed using NanoString’s nSolver Analysis Software v4.0 (https://www.nanostring.com; NanoString Technologies, Seattle, WA, USA) with raw counts normalized using house-keeping genes, selected based on a low coefficient of variation between samples and average counts above negative controls. Differential gene expression was derived using nCounter default settings and quality control governed as per manufacturer’s instructions.

### 4.7. Whole-Genome Sequencing (WGS) and Bioinformatics Analysis Pipeline

WGS was performed on an Illumina HiSeqX instruments (Illumina, San Diego, CA, USA) by Genome.One Pty Ltd. (Sydney, NSW, Australia) and at Macrogen (Seoul, Korea). Germline DNA (blood) was sequenced to a depth 30–45X and the experimental samples to 60–90X depending on the sequencing provider.

Using a Burrows–Wheeler Aligner (BWA-MEM v0.7.10-r789) [54], paired-end sequencing reads were aligned to Genome Reference Consortium Human Build 37 (GRCh37/hg19) and improved using realignment around known indels using the Genome Analyser toolkit (GATK) version 3.3.0. PCR duplicates were removed using SAMtools v1 and Picard metrics have been used to evaluate the run quality. Somatic single nucleotide variants (SNVs) were called by Strelka v1.0.15 [55] from tumor-normal pairs. Calculations of the tumor cellularity, ploidy, and copy number alterations were performed by Purple and Sequenza 2.1.2 [56,57]. Major structural variants (SVs) were inferred with Manta 0.27.1 [58]. The annotation and further filtering of Strelka quality-passed SNVs and indels were done based on two different platforms, i.e., OpenCravat [59] and Gemini-Seave pipeline [60]. Mutational signatures were determined for each specimen as per the method described by Mueller et al. [18]. Using the Integrated Genomics Viewer (IGV; Broad Institute, Cambridge, MA, USA) somatic variants of interest were verified to ensure coverage and call accuracy. Unique and shared somatic mutation sets were obtained for each sequenced sample using Bedtools v2.27.0.

### 4.8. Chemical Compound Library Assays and Analysis

Cells were screened using the CHiP 2G anti-cancer and kinase-inhibitor libraries (SelleckChem, Houston, TX, USA), at a concentration of 1 µM. A total of 1000 cells were seeded per well into 384-well plates (PerkinElmer, Waltham, MA, USA, Cat. no. 6007558). Cells were cultured at 37 °C, 5% CO_2_ and treated with anti-cancer (Selleckchem, Houston, TX, USA, Cat. no. L3000) and anti-kinase (Selleckchem, USA, Cat. no L1200) small molecule libraries 24 h after cell seeding. Cell viability was measured after 72 h using the CellTiter-Glo^®^ Luminescent assay (Promega, Madison, WI, USA, Cat no. G7570) as per manufacturer’s protocol. Percentage viability was calculated from replicates relative to the average DMSO control reading.

### 4.9. Secondary Dose-Response Screening

Based on clinical use and the results of the high-throughput screen, cell lines were screened with a suite of chemotherapeutic agents including PIK-75 (Selleckchem, USA, Cat no. S1205) at a titrated range. The metabolic activity of cells was determined using a CellTitre 96^®^ Aqueous One Solution Cell Proliferation Assay (Promega, USA, Cat no. G3581) according to the manufacturer’s instructions and as previously described [61]. The raw data of treated cells were normalized against vehicle controls with background absorbance values subtracted. Half-maximal inhibitory concentration (IC_50_) values were derived with GraphPad Prism 6.0 using a logarithmic sigmoidal dose–response curve with the variable slope parameter. The cell viability of treated cells was normalized against vehicle controls.

### 4.10. Cell Lysate Preparation and Western Blot Analysis

Near-confluent UW-CSCC1 and UW-CSCC1-R were treated with IC_50_ concentrations of PIK-75 (120 nM or 200 nM, respectively) or dactolisib (22 nM or 80 nM, respectively) for 3 and 6 h, along with a 6 h DMSO vehicle control treatment. Lysates were prepared by washing the cells in PBS, followed by a 20 min incubation with RIPA buffer (50 mM Tris-HCl (pH = 7.4), 150 mM NaCl, 1% Triton-x100, 5 mM EDTA, 1 mM PMSF, 1 mM sodium orthovandate) on ice. The crude lysate was transferred to a microfuge tube and centrifuged at 12,000× *g* for 5 min at 4 °C to pellet debris and genomic DNA. The proteinaceous supernatant was subsequently aliquoted for long-term storage at −80 °C. The protein concentration of lysates was determined using a Pierce Bicinchoninic Acid (BCA) Protein Assay Kit (ThemoFisher Scientific, Waltham, MA, USA) according to the manufacturer’s protocol.

Protein extracts (20 µg) were resolved on a 4–20% gradient SDS-polyacrylamide gel (Invitrogen, Carlsbad, CA, USA, Cat no. NP0324BOX) under reducing conditions. Proteins were transferred to a PVDF membrane and blocked in 5% skim milk powder containing Tris-buffered saline with 0.2% tween (TBST) for one hour at room temperature. Membranes were rinsed with TBST and then incubated overnight at 4 °C with primary antibodies from Cell Signalling (Danvers, MA, USA), including: rabbit anti-human AKT ([11E7] 1:1000, Cat no. 46855), rabbit anti-human phospho-AKT (Ser473; D9E, 1:1000, Cat no. 4060S), and the housekeeping mouse anti-human GAPDH (D4C6R, 1:2000, Cat no. 97166S) diluted in TBST containing 2% skim milk powder. After three rinses with TBST, membranes were incubated for 90 min at room temperature with horseradish peroxidase-conjugated anti-rabbit (Cell Signalling, Danvers, MA, USA, Cat no. 7074), and anti-mouse (Abcam, Cambridge, UK, Cat no. ab205719) IgG secondary antibodies at a dilution of 1:2000 in TBST containing 2% skim milk powder. Chemiluminescence was generated using Pierce™ ECL Western Blotting Substrate (ThermoFisher Scientific, Waltham, MA, USA, Cat no. 32109) and visualized on the Amersham™ Imager 600 (GE Healthcare, Chicago, IL, USA). Captured blots were subsequently analyzed using ImageJ (version 1.53) to obtain densitometry. The resulting densitometry units were then graphically interpreted using GraphPad Prism5 v6.0.

### 4.11. Data Repository

The variant call format files have been deposited at the European Genome-Phenome Archive (https://ega-archive.org/), which is hosted by the EMBL-European Bioinformatics Institute (Cambridgeshire, UK) and the Center for Genomic Regulation (Barcelona, Spain), under accession number EGAD00001004530.

## Figures and Tables

**Table 1:** IC_50_ values of chemotherapeutic agents against UW-CSCC1 and UW-CSCC2. Compounds are listed in order of lowest IC_50_ presented either as a mean or mean ± standard error of the mean (SEM) from > 2 independent experiments each performed in triplicate.

Compound	Drug Class	UW-CSCC1	UW-CSCC1-R	UW-CSCC2
		IC_50_ µM		
Dactosilib	Dual PI3K/mTOR inhibitor	0.022 ± 0.003	0.080 ± 0.023	0.028 ± 0.012
PIK-75	PI3K inhibitor	0.122 ± 0.012	0.204 ± 0.05	0.026 ± 0.012
5-Fluorouracil	Antimetabolite	4.47 ± 0.4	7.56 ± 0.1	-
Carboplatin ^†^	Platinum analogue	22.40	22.90	200.00

^†^ Compound not present in HTS drug screen.

## Supplementary Materials

The following are available online at https://www.mdpi.com/1422-0067/21/24/9536/s1.

## Abbreviations

cSCC	Cutaneous squamous cell carcinoma
NER	Nuclear excision repair
SNV	Small nucleotide variant
UV	Ultraviolet radiation
WGS	Whole genome sequencing
