# Supplementary material for: Comprehensive Mutational and Phenotypic Characterization of New Metastatic Cutaneous Squamous Cell Carcinoma Cell Lines Reveal Novel Drug Susceptibilities
Source: Int J Mol Sci. 2020 Dec 15;21(24):9536. doi: 10.3390/ijms21249536 (PMC7765308; doi:10.3390/ijms21249536)
Supplement: Supplementary file 1 [file ijms-21-09536-s001.zip › ijms-1006243-supporting/ijms-1006243-supporting information.pdf]

SUPPLEMENTARY MATERIALS AND METHODS

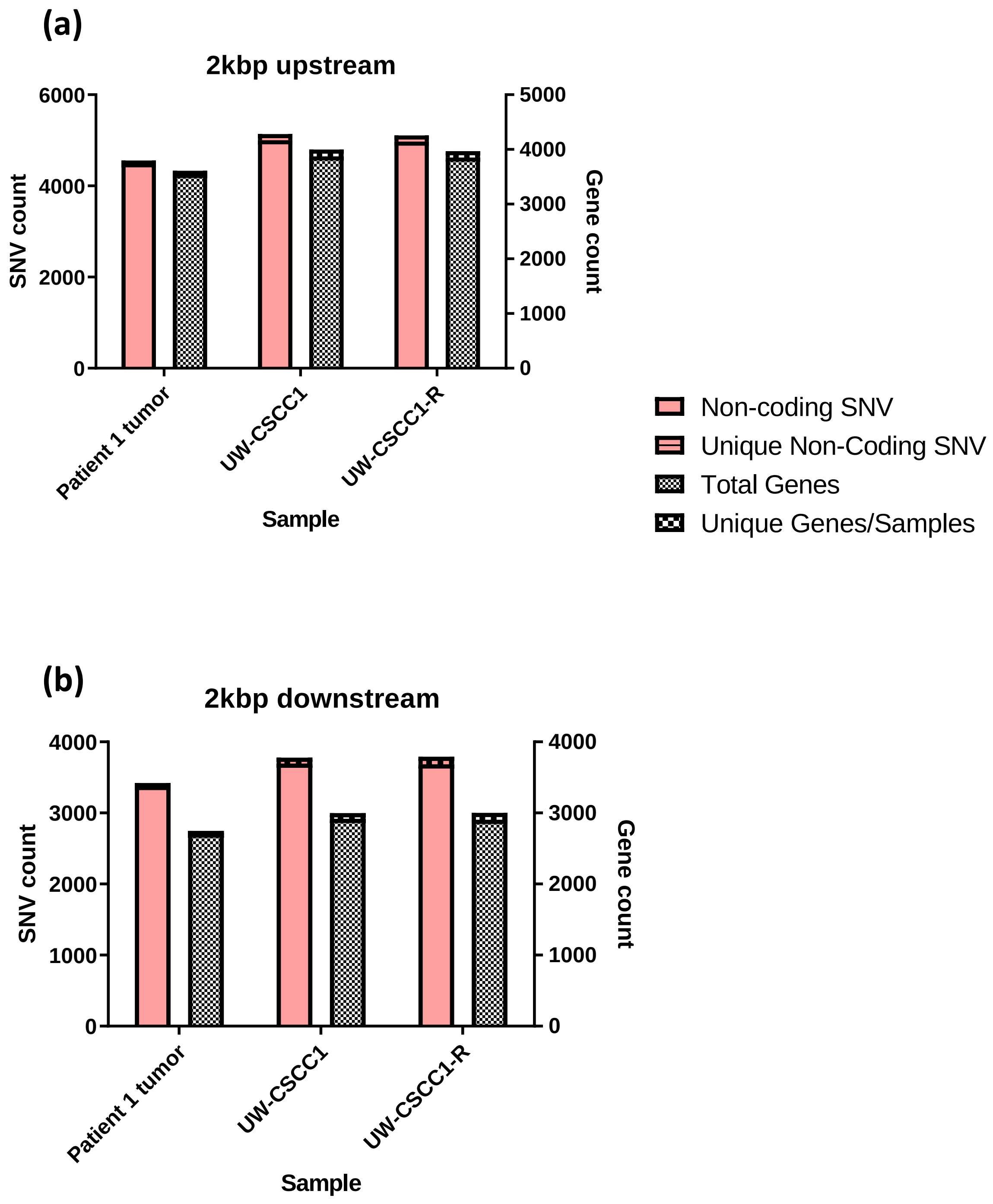

Figure S1. The degree of non-coding SNVs in the 2kbp (**a**) upstream and (**b**) downstream (b) region. The total number of non-coding SNVs found in these regions shared between the samples are shown stacked with the number of non-coding SNVs unique to each sample. Additionally, the total number of genes impacted by

non-coding SNVs are shown stacked with the number of genes carrying an SNV unique to that sample.

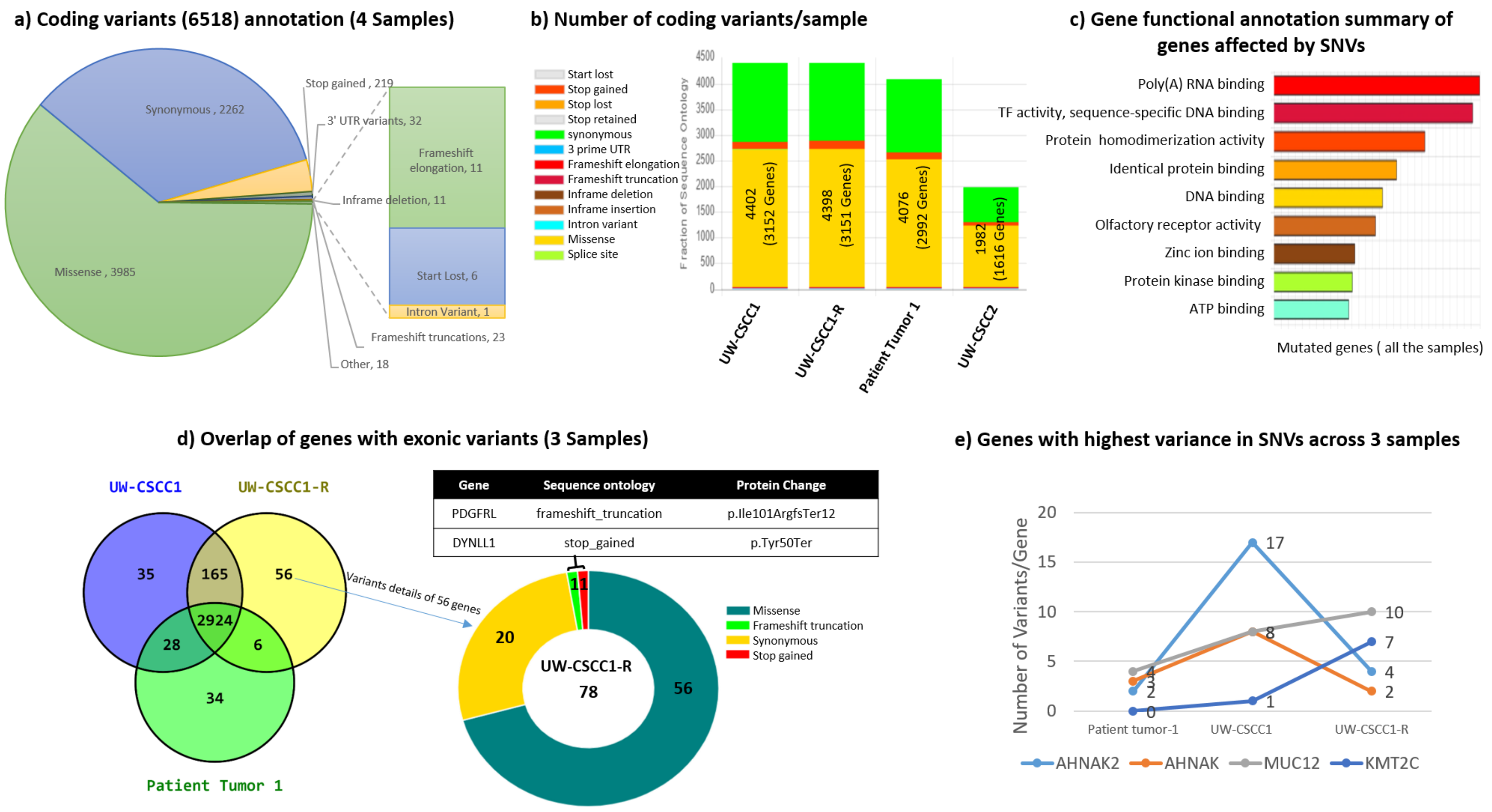

Figure S2. Exonic variants summary. (**a**) A total of 6518 variants were detected across 4 samples (i.e. Patient tumor 1, UW-CSCC1, UOW-CSCC1-R and UW-CSCC2), most of them being missense and synonymous. (**b**) Values in each bar represent the number of the variants and corresponding impacted genes. This was high for Patient tumor 1 and associated cell lines, but significantly lower for the UW-CSCC2 cell line. (**c**) Shows the annotation of the genes affected by mutations. Most of them relate to poly(A) RNA binding and Transcription factor (TF) activity sequence-specific DNA binding (**d**) Most of the genes (~98%) and coding variants are shared between Patient tumor 1 and associated cell lines (number of shared variants are not shown). 56 new unique genes were found in UW-CSCC1-R with 78 mutations and, as shown in the figure, 2 genes have frameshift and stop gained SNVs. (**e**) From the

list of genes from 3 samples (Patient tumor 1 & associated cell-lines) having >=6 variants in any sample, 4 genes (*AHNAK2*, *AHNAK,* *MUC12*, and *KMT2C*) had very high variance among these samples. *AHNAK*/*AHNAK2* is suspected to regulate PI3K signalling and migration; *MUC12* is involved in epithelial cell adhesion and signalling; *KMT2C* is involved in histone methylation activity.

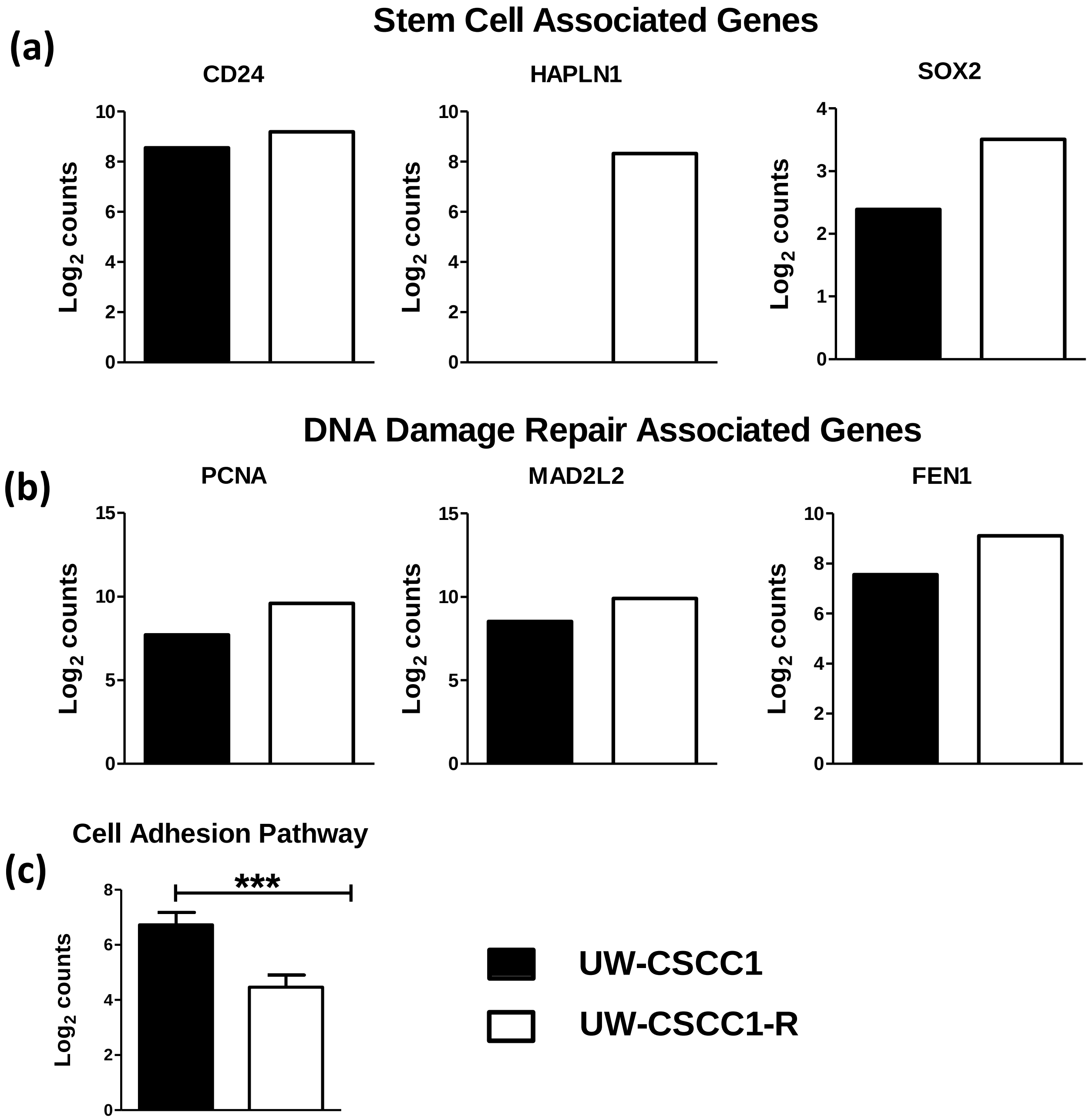

Figure S3. Expression of cell adhesion pathway and stem cell associated genes between UW-CSCC1 and UW-CSCC1-R. **a**) Log_2 normalised counts for stem cell associated genes: *CD24*, *SOX2*, and *HAPLN1*. (**b**) Log_2 normalised counts for DNA damage repair associated genes: *PCNA, MAD2L2*, and *FEN1*. (**c**) Log_2 normalised counts of the pathway score for cell adhesion genes. Error bars refer to standard error of the mean, n = 56 genes. Asterisks denote $p < 0.001$ calculated using an unpaired student's t-test.

Table S1. Reported cell lines derived from cSCC lymph node metastases. Tumour, node, metastasis (TNM) staging in accordance to the 7th AJCC cancer staging manual. WD: well differentiated; MD: moderately differentiated; PD: poorly differentiated.

Cell Line	Primary location	TNM	Histological Grade	Reference
UT-SCC-7	Temporal skin	T1N0M0	WD	[1]
UT-SCC59A	Temporal skin	T1N3M0	MD	[2]
UT-SCC115	Auricular skin	T2N2aM0	MD	[2]
Undesignated	Parietal skin	T2N0M0	PD	[3]
MET4	Skin of hand	NA	NA	[4]
IC1MET	Temporal skin	NA	NA	[5]

Table S2. Clinicopathologic details of metastatic cSCC patients.

Sample	Patient 1 (UW-CSCC1)	Patient 2 (UW-CSCC2)
**% tumour cellularity***	70/34	<10/14
**Primary location**	Unknown	Temporal scalp
**Metastasis location**	Right parotid	Right parotid
**Degree of differentiation**	Poorly differentiated	Moderately differentiated
**Age at resection**	73	86
**Sex**	M	M
**TNM**	TxN3bM0	TxN2bM0
**Immunosuppression**	N	N
**Adjuvant radiotherapy**	Y	Y
**Specimen ID**	CSCC_0003	CSCC_0031

*% purity - as per pathologist assessment/ as defined by Purple

Table S3. List of genes derived from analysis of recurrent coding short variants in known oncogenes, tumor suppressor genes and other driver genes (*n* = 1309) in 15 metastatic cSCC patient specimens (Mueller et al 2019; Ashford et al submitted) shared by UW-CSCC1/-R and UW-CSCC2). N=60 genes. * Refers to the proportion of patient specimens with coding variants (no impact level prescribed) out of the total specimen cohort of 15.

Gene	No. Specimens /15*	Gene	No. Specimens /15*
AHNAK2	15	*KALRN*	12
AKT3	4	*KIT*	7
AMPH	9	*KMT2D*	13
BAI3	12	*KRT19*	2
CD163	8	*MACF1*	11
CDH11	7	*MDN1*	11
CNBD1	13	*MECOM*	7
COL1A2	13	*MKI67*	6
COL4A2	11	*MUC17*	12
COL5A3	12	*NBEA*	13
COL6A1	8	*NEB*	13
COL6A2	9	*NLRP1*	4
COL6A3	13	*NLRP3*	8
CUX1 (Patient tumor 1 and UW-CSCC2 only)	11	*NOTCH1*	13
DCC	12	*NR4A1*	5
DNAH12	10	*NRXN1*	11
EPHA1	3	*NSD1*	7
EPS8L1	5	*PIK3CG*	11
ERBB3	6	*PLEC*	14
ERBB4	8	*PTPRC*	8
FBN1	11	*PTPRM*	8
FLT1	7	*RNF213*	10
FN1	13	*SULF1*	5
FREM1	9	*SYNE1*	14
FREM2	10	*TIE1*	9
HK3	9	*TNXB*	13
INTS1	13	*TP53*	13
ITGA5	6	*VCAN*	12
ITGAV	4	*WHSC1*	7
ITGB3	8	*XIRP2*	13

Table S4. Copy number alterations for PI3K/mTOR associated genes between Patient 1 tumor and the cell lines. Copy number determined using Sequenza, with a minimum copy number variation of four used as the cutoff for the patient 1 tumor.

Gene	Sample	Copy Number	Event Type	Method	Block Coordinates	Block Size (bp)	CN.A	CN.B	AF.B	DP.ratio
PIK3CD	UW-CSCC1	3	duplication	Sequenza	1:5737081-17193647	11456566	2	1	0.333790064	0.908720155
PIK3CD	Patient 1 tumor	4	duplication	Sequenza	1:8382403-12923209	4540806	2	2	0.427127922	1.00145165
PIK3CD	UW-CSCC1-R	3	duplication	Sequenza	1:5877284-11386625	5509341	2	1	0.327109315	0.950606773
PIK3CD	UW-CSCC2	20	duplication	Sequenza	1:9471651-9822331	350680	20	0	0.00095387	0.85279981
MTOR	UW-CSCC1	3	duplication	Sequenza	1:5737081-17193647	11456566	2	1	0.333790064	0.908720155
MTOR	Patient 1 tumor	4	duplication	Sequenza	1:8382403-12923209	4540806	2	2	0.427127922	1.00145165
MTOR	UW-CSCC1-R	3	duplication	Sequenza	1:5877284-11386625	5509341	2	1	0.327109315	0.950606773
MTOR	UW-CSCC2	20	duplication	Sequenza	1:10898539-11581720	683181	20	0	0.00130281	0.81969113
HDAC1	UW-CSCC1	3	duplication	Sequenza	1:17276627-41979243	24702616	2	1	0.332362384	0.91326459
HDAC1	Patient 1 tumor	4	duplication	Sequenza	1:31128555-41979243	10850688	2	2	0.430630585	0.995660518
HDAC1	UW-CSCC1-R	3	duplication	Sequenza	1:31387701-37026270	5638569	2	1	0.327131117	0.954404968
PIK3CB	UW-CSCC1	4	duplication	Sequenza	3:133136733-140431565	7294832	2	2	0.447644804	1.148496054
PIK3CB	Patient 1 tumor	4	duplication	Sequenza	3:133142760-140621480	7478720	2	2	0.44822753	1.06563704
PIK3CB	UW-CSCC1-R	4	duplication	Sequenza	3:137478795-140431565	2952770	2	2	0.46237796	1.240440398
PIK3CA	UW-CSCC1	5	duplication	Sequenza	3:176561143-180717797	4156654	3	2	0.408984733	1.332820632
PIK3CA	Patient 1 tumor	4	duplication	Sequenza	3:178849290-179057440	208150	2	2	0.4505252	1.083533554
PIK3CA	UW-CSCC1-R	5	duplication	Sequenza	3:178844813-180717797	1872984	3	2	0.39663617	1.500260047
PIK3CG	UW-CSCC2	6	duplication	Sequenza	7:106279626-108309540	2029914	3	3	0.45120407	0.97910388
NFKB1	UW-CSCC1	3	duplication	Sequenza	4:92040827-117022057	24981230	2	1	0.334089773	0.845295731
NFKB1	Patient 1 tumor	3	duplication	Sequenza	4:52660855-123863411	71202556	2	1	0.386054905	0.970871385
HDAC3	UW-CSCC1	3	duplication	Sequenza	5:140584278-141131457	547179	3	0	0.001276206	0.9156575
HDAC3	Patient 1 tumor	3	duplication	Sequenza	5:119108766-180904762	61795996	3	0	0.281487242	0.914362486
HDAC3	UW-CSCC1-R	3	duplication	Sequenza	5:140103029-141226970	1123941	3	0	0.000130453	0.926647872
HDAC2	UW-CSCC1	4	duplication	Sequenza	6:94543293-119802083	25258790	3	1	0.241703655	1.16090065
HDAC2	Patient 1 tumor	5	duplication	Sequenza	6:100808361-119799490	18991129	4	1	0.323483789	1.17299583
HDAC2	UW-CSCC1-R	4	duplication	Sequenza	6:109371129-115330657	5959528	3	1	0.251406996	1.177661485

Table S5. Samples run on the NanoString nCounter system. The passage number the nucleic acid was extracted from for each cell line is shown. For some UW-CSCC1 derivatives, passage numbering started after inception of the derivative. i.e. UW-CSCC1 normoxic was analyzed after 3 subcultures in a normoxic atmosphere from UW-CSCC1 passage 13, thereby gaining the designation 13.03. Passage 13 and 4 are the earliest passage at which a pure culture of epithelial cells was confirmed for UW-CSCC1 and UW-CSCC2, respectively. The panel used for each sample (PanCancer Progression and/or PanCancer Pathways) is shown.

Sample	Passage Number	Panel used
UW-CSCC1 (low passage)	13	Progression/Pathways
UW-CSCC1 High passage	41	Progression/Pathways
UW-CSCC1 Normoxic	13.03	Progression
UW-CSCC1 Spheroid	13.05	Progression/Pathways
UW-CSCC1 Xenograft	-	Progression/Pathways
UW-CSCC1 Xenograft secondary cell line	2	Progression
UW-CSCC1-R	13.08	Progression/Pathways
UW-CSCC2	4	Progression
Patient 1 tumor (origin of UW-CSCC1)	-	Progression/Pathways

Table S6.1. Top 40 compounds from anti-cancer library against UW-CSCC1. All tested at 1 μM.

Compound	UW-CSCC1	Pathway	Target
Flavopiridol (Alvocidib)	98.2	Cell Cycle	CDK
Daunorubicin HCl	97.61	DNA Damage	Telomerase
Elesclomol	97.6	Angiogenesis	HSP
MLN2238	97.18	Proteases	Proteasome
Bortezomib (Velcade)	97.15	Proteases	Proteasome
Topotecan HCl	96.98	DNA Damage	Topoisomerase
PIK-75	96.96	PI3K/Akt/mTOR	DNA-PK, PI3K
Flavopiridol (Alvocidib) HCl	96.69	Cell Cycle	CDK
MLN9708	96.66	Proteases	Proteasome
SNS-032 (BMS-387032)	96.53	Other	CDK
Doxorubicin (Adriamycin)	95.71	DNA Damage	Topoisomerase
Triptolide	95.48	Other	Other
Gemcitabine (Gemzar)	95.3	DNA Damage	Other
Clofarabine	94.78	DNA Damage	DNA/RNA Synthesis
Disulfiram (Antabuse)	94.68	Other	Other
Ganetespib (STA-9090)	94.52	Other	HSP
Romidepsin	94.32	Cytoskeletal Signalling	HDAC
Mitoxantrone HCl	94.23	Other	Other
MK-1775	94.09	Cell Cycle	Wee1
Mitoxantrone	94.03	Cell Cycle	Topoisomerase
HSP990 (NVP-HSP990)	93.8	Cytoskeletal Signalling	HSP (e.g. HSP90)
17-DMAG HCl	93.46	Other	HSP
AUY922 (NVP-AUY922)	93.16	Other	HSP
Teniposide (Vumon)	93.04	Other	Other
Vinorelbine Tartrate	92.76	Cytoskeletal Signalling	Microtubule Associated
AZD7762	92.63	Cell Cycle	Chk
JNJ-26481585	91.97	Other	HDAC
Geldanamycin	91.58	Cytoskeletal Signalling	HSP
BIIB021	90.39	Cytoskeletal Signalling	HSP
AT7519 HCl	90.34	Cell Cycle	CDK
Torin 2	89.92	PI3K/Akt/mTOR	mTOR
Vincristine	89.39	Cytoskeletal Signalling	Autophagy, Microtubule Associated
AT7519	89.29	Cell Cycle	CDK
Epothilone A	88.56	Cytoskeletal Signalling	Microtubule Associated
VER-50589	88.24	Cytoskeletal Signalling	HSP (e.g. HSP90)
Plinabulin (NPI-2358)	87.24	Angiogenesis	VDA
Obatoclax mesylate	87.09	Neuronal Signalling	Bcl-2
KX2-391	87.07	Angiogenesis	Src
Docetaxel (Taxotere)	87.01	Other	Microtubule Associated
Trichostatin A (TSA)	86.88	Other	HDAC

Table 6.2. Top 40 compounds from anti-cancer library against UW-CSCC1-R. All tested at 1 μM.

Compound	UW-CSCC1-R	Pathway	Target
Flavopiridol (Alvocidib)	98.32	Cell Cycle	CDK
MLN9708	98.28	Proteases	Proteasome
AZD7762	97.95	Cell Cycle	Chk
Daunorubicin HCl	97.89	DNA Damage	Telomerase
MLN2238	97.66	Proteases	Proteasome
PIK-75	97.55	PI3K/Akt/mTOR	DNA-PK, PI3K
Elesclomol	97.47	Angiogenesis	HSP
Bortezomib (Velcade)	97.41	Proteases	Proteasome
Clofarabine	97.00	DNA Damage	DNA/RNA Synthesis
Flavopiridol HCl	96.96	Cell Cycle	CDK
Topotecan HCl	96.88	DNA Damage	Topoisomerase
Doxorubicin (Adriamycin)	96.84	DNA Damage	Topoisomerase
MK-1775	96.72	Cell Cycle	Wee1
Gemcitabine (Gemzar)	96.61	DNA Damage	Other
Vinorelbine Tartrate	95.99	Cytoskeletal Signalling	Microtubule Associated
LY2603618 (IC-83)	95.93	Cell Cycle	Chk
Teniposide (Vumon)	95.88	Other	Other
Mitoxantrone HCl	94.96	Other	Other
Triptolide	94.65	Other	Other
CYT997 (Lexibulin)	94.24	Cytoskeletal Signalling	Microtubule Associated
Mitoxantrone	94.06	Cell Cycle	Topoisomerase
SNS-032 (BMS-387032)	94.01	Other	CDK
SF1670	93.94	Other	Other
Romidepsin	93.74	Cytoskeletal Signalling	HDAC
JNJ-26481585	93.64	Other	HDAC
Torin 2	93.57	PI3K/Akt/mTOR	mTOR
Epothilone A	93.48	Cytoskeletal Signalling	Microtubule Associated
Ganetespib (STA-9090)	93.18	Other	HSP
BIIB021	92.58	Cytoskeletal Signalling	HSP
CH5138303	92.44	Cytoskeletal Signalling	HSP (e.g. HSP90)
Vincristine	92.32	Cytoskeletal Signalling	Autophagy, Microtubule Associated
PU-H71	92.1	Cytoskeletal Signalling	HSP
Voreloxin (SNS-595)	91.96	DNA Damage	Topoisomerase
17-DMAG HCl	91.88	Other	HSP
Obatoclax mesylate	91.67	Neuronal Signalling	Bcl-2
Docetaxel (Taxotere)	91.47	Other	Microtubule Associated
KX2-391	91.28	Angiogenesis	Src
HSP990 (NVP-HSP990)	91.27	Cytoskeletal Signalling	HSP (e.g. HSP90)
Disulfiram (Antabuse)	90.85	Other	Other
Plinabulin (NPI-2358)	90.69	Angiogenesis	VDA

Table 6.3. Top 40 compounds from kinase-inhibitor library against UW-CSCC1. All tested at 1 μM.

Compound	UW-CSCC1	Pathway	Target
PIK-75	97.85	PI3K/Akt/mTOR	DNA-PK, PI3K
BGT226 (NVP-BGT226)	97.62	PI3K/Akt/mTOR	mTOR, PI3K
Dinaciclib (SCH727965)	96.02	Cell Cycle	CDK
SNS-032 (BMS-387032)	95.9	Other	CDK
Flavopiridol HCl	95.63	Cell Cycle	CDK
Staurosporine	94.9	TGFO-beta/Smad	PKC
CUDC-907	94.81	Cytoskeletal Signalling	HDAC, PI3K
Flavopiridol (Alvocidib)	93.6	Cell Cycle	CDK
AZD7762	89.55	Cell Cycle	Chk
KX2-391	88.63	Angiogenesis	Src
Torin 2	85.43	PI3K/Akt/mTOR	mTOR
Hesperadin	81.4	Cell Cycle	Aurora Kinase
CHIR-124	77.59	Cell Cycle	Chk
AT7519	77.38	Cell Cycle	CDK
VE-822	77.22	PI3K/Akt/mTOR	ATM/ATR
Rigosertib (ON-01910)	76.97	Cell Cycle	PLK
PHA-793887	74.79	Cell Cycle	CDK
FIIN-2	73.99	Protein Tyrosine Kinase	FGFR
JNK Inhibitor IX	72.68	MAPK	JNK
PF-3758309	72.26	Cytoskeletal Signalling	PAK
LY2603618	70.49	Cell Cycle	Chk
Mubritinib (TAK 165)	69.62	Protein Tyrosine Kinase	HER2
PF-477736	69.23	Cell Cycle	Chk
Ro3280	68.26	Cell Cycle	PLK
INK 128 (MLN0128)	66.91	PI3K/Akt/mTOR	mTOR
Tivantinib (ARQ 197)	66.52	Protein Tyrosine Kinase	c-Met
AZD8055	66.27	PI3K/Akt/mTOR	mTOR
ENMD-2076	65.96	Angiogenesis	Aurora Kinase, Flt, VEGFR
Trametinib	65.78	MAPK	MEK
GSK1059615	64.27	PI3K/Akt/mTOR	mTOR, PI3K
GSK461364	64.18	Cell Cycle	PLK
PF-04691502	61.01	PI3K/Akt/mTOR	Akt, mTOR, PI3K
PD173074	60.98	Angiogenesis	FGFR, VEGFR
CX-6258 HCl	60.63	JAK/STAT	Pim
AZD2014	60.27	PI3K/Akt/mTOR	mTOR
Tyrphostin 9	58.58	Protein Tyrosine Kinase	EGFR
WYE-125132	56.76	PI3K/Akt/mTOR	mTOR
Volasertib (BI 6727)	56.44	Cell Cycle	PLK
CCT137690	56.38	Cell Cycle	Aurora Kinase
Dasatinib	56.15	Angiogenesis	Bcr-Abl, c-Kit, Src

Table 6.4. Top 40 compounds from kinase-inhibitor library against UW-CSCC1-R. All tested at 1 μM.

Compound	UW-CSCC1-R	Pathway	Target
PIK-75	98.43	PI3K/Akt/mTOR	DNA-PK, PI3K
SNS-032 (BMS-387032)	97.94	Other	CDK
BGT226 (NVP-BGT226)	97.84	PI3K/Akt/mTOR	mTOR, PI3K
VE-822	97.36	PI3K/Akt/mTOR	ATM/ATR
Dinaciclib (SCH727965)	97.17	Cell Cycle	CDK
LY2603618	97.07	Cell Cycle	Chk
CHIR-124	96.93	Cell Cycle	Chk
Flavopiridol HCl	96.75	Cell Cycle	CDK
AZD7762	96.64	Cell Cycle	Chk
Staurosporine	96.42	TGFO-beta/Smad	PKC
Flavopiridol (Alvocidib)	95.26	Cell Cycle	CDK
CUDC-907	95.15	Cytoskeletal Signalling	HDAC, PI3K
KX2-391	95.09	Angiogenesis	Src
PF-477736	94.94	Cell Cycle	Chk
MK-8776 (SCH 900776)	92.22	Cell Cycle	CHK
Torin 2	91.96	PI3K/Akt/mTOR	mTOR
AZ20	90.96	PI3K/Akt/mTOR	ATM/ATR
PF-3758309	90.13	Cytoskeletal Signalling	PAK
GSK461364	90	Cell Cycle	PLK
Ro3280	88.91	Cell Cycle	PLK
AP26113	88.61	Protein Tyrosine Kinase	ALK
Hesperadin	86.63	Cell Cycle	Aurora Kinase
JNK Inhibitor IX	85.9	MAPK	JNK
CX-6258 HCl	85.01	JAK/STAT	Pim
Tivantinib (ARQ 197)	83	Protein Tyrosine Kinase	c-Met
Rigosertib (ON-01910)	81.59	Cell Cycle	PLK
R428 (BGB324)	81.48	Protein Tyrosine Kinase	Other
BI 2536	78.72	Other	PLK
Volasertib (BI 6727)	76.4	Cell Cycle	PLK
Mubritinib (TAK 165)	75.15	Protein Tyrosine Kinase	HER2
Milciclib (PHA-848125)	74.7	Cell Cycle	CDK
AZ 960	72.11	JAK/STAT	JAK
INK 128 (MLN0128)	68	PI3K/Akt/mTOR	mTOR
GSK1059615	67.63	PI3K/Akt/mTOR	mTOR, PI3K
Tyrphostin 9	66.39	Protein Tyrosine Kinase	EGFR
AZD6738	66.15	PI3K/Akt/mTOR	ATM/ATR
Pelitinib (EKB-569)	65.6	Protein Tyrosine Kinase	EGFR
AZD8055	59.52	PI3K/Akt/mTOR	mTOR
PF-04691502	58.26	PI3K/Akt/mTOR	Akt, mTOR, PI3K
SNS-314 Mesylate	58.16	Other	Aurora Kinase

Supplementary References

1. Pekkola-Heino, K., Kulmala, J., Grenman, R., *Sublethal damage repair in squamous cell carcinoma cell lines.* Head & Neck, 1992. **14**: p. 196-199.
2. Farshchian, M., et al., *Serpin Peptidase Inhibitor Clade A Member 1 (SerpinA1) Is a Novel Biomarker for Progression of Cutaneous Squamous Cell Carcinoma.* The American Journal of Pathology, 2011. **179**(3): p. 1110-1119.
3. Anderson, A.N., et al., *Functional genomic analysis identifies drug targetable pathways in invasive and metastatic cutaneous squamous cell carcinoma.* 2020. **6**(4).
4. Proby, C.M., et al., *Spontaneous keratinocyte cell lines representing early and advanced stages of malignant transformation of the epidermis.* Experimental dermatology, 2000. **9**(2): p. 104-17.
5. Inman, G.J., et al., *The genomic landscape of cutaneous SCC reveals drivers and a novel azathioprine associated mutational signature.* Nature Communications, 2018. **9**(1): p. 3667.
